# Gestational diabetes mellitus and labor analgesia: nationwide register-based analysis in Finland

**DOI:** 10.1007/s00592-022-01944-6

**Published:** 2022-08-02

**Authors:** Ilari Kuitunen, Sakari Vähä-Tuisku, Tuomas Huttunen

**Affiliations:** 1 Institute of Clinical Medicine and Department of Pediatrics, University of Eastern Finland, Kuopio, Finland; 2 Department of Pediatrics, Mikkeli Central Hospital, Porrassalmenkatu 35-37, 50100 Mikkeli, Finland; 3 Department of Anesthesia, Päijät-Häme Central Hospital, Lahti, Finland; 4 Faculty of Medicine and Health Technologies, Tampere University, Tampere, Finland; 5 Department of Anesthesia and Intensive Care, Tampere Heart Hospital, Tampere University Hospital, Tampere, Finland

**Keywords:** Gestational diabetes mallitus, Analgesia, Epidural, Spinal

## Short communication

Diabetes Mellitus (DM) has been shown to have an effect on patient analgesia, as DM patients often require higher doses and longer courses of postoperative analgesia [1]. The effect of Gestational DM (GDM) on labor analgesia has not been studied well and for example National Institute for Health and Care Excellence has no recommendations regarding the labor analgesia of GDM patients [2]. Only one previous study has assessed labor analgesia in parturients with GDM and they reported that opioid consumption was higher after cesarean section in parturients with GDM [3]. GDM parturients are usually more obese than non-GDM parturients. A previous register analysis showed that there were little differences in the use of labor analgesia based on the grade of obesity [4]. As the incidence of GDM is still increasing, it is important to know whether it influences the need of labor analgesia [5].

The aim of our study is to analyze labor analgesia between GDM and non-GDM parturients on a national level.

## Materials

We conducted a nationwide retrospective cohort study. We used the data from Finnish Medical Birth Register, which is maintained by the Finnish Institute of Health and Welfare. The coverage of the register is nearly 100%, and it includes all pregnancies ending in delivery after gestational week 21 or fetal weight of 500 g or more. For this study, we included all deliveries from 2004 to 2018 where the GDM status was known. GDM testing results have been reported to the register since 2004. In Finland, GDM is tested by 2 h oral glucose tolerance test (OGTT) (75 g of glucose) during the second trimester. Pathologic OGTT findings (1-h glucose > 10.0 mmol/l or 2-h glucose value > 8.6 mmol/l) are diagnosed as GDM and reported to the register. In some cases (for example fetal macrosomia), the test can be reperformed later in pregnancy. Women with young age (less than 25 years), normal BMI (18–25 kg/m^2^) do not need to undergo the OGTT.

Our main outcome was the use of labor analgesia. The analgesia methods were stratified into neuraxial analgesia (epidural, spinal and combined), pudendal, paracervical, nitrous oxide, other medical, other non-medical and no analgesia. These are analyzed as categorized (yes or no) variables, as the register does not contain more precise information for example on the dosage used. We calculated the yearly amount of performed OGTTs, from which we separated normal and pathological results in our included study population.

No ethical committee evaluation was needed due to the register-based retrospective study design. Our study has the research permission from the Finnish data authority Findata, which was granted after critical evaluation of our study protocol.

## Results

**Fig. 1:**
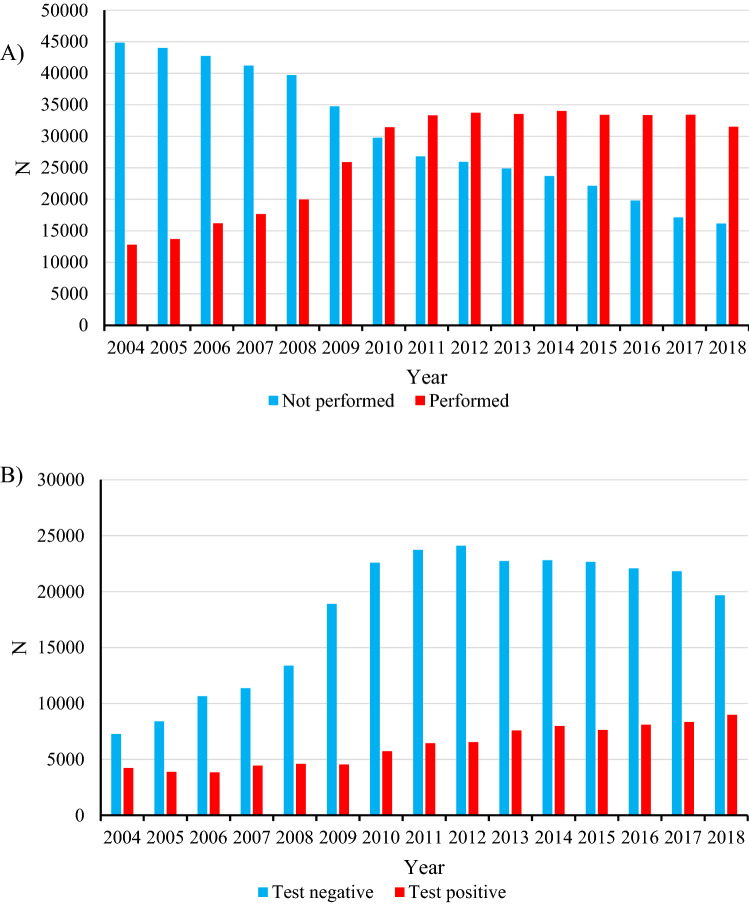
**a** Yearly number of parturients for who oral glucose tolerance test was performed and not performed in Finland from 2004 to 2018. **b** Yearly numbers of negative OGTT results and positive GDM findings in Finland from 2004 to 2018

We retrieved a total of 857,578 pregnancies from the register. The number of pregnancies where OGTT was not performed has been decreasing continuously since 2004, whereas the number of pregnancies with OGTT first increased and have then remained stable since 2011 (Fig. 1a).

For labor analgesia analysis, we excluded pregnancies without OGTT (*n* = 453,630), non-singleton pregnancies (*n* = 11,705), elective cesarean sections (*n* = 26,362), and out-of-hospital deliveries (*n* = 764). Altogether 365,115 deliveries were included in the final analysis. The GDM group included 92,929 (25.5%) deliveries and the non-GDM group 272,186 (74.5%) deliveries. The diagnosis of GDM increased steadily throughout the study period (Fig. 1B).

Neuraxial analgesia was used in 67.2% of labors in the GDM group and 67.9% in the non-GDM group. A slight difference was seen in the rate of epidural analgesia (47.8% in GDM group and 49.9% in non-GDM group, Table 1). The use of gynecological local (pudendal and paracervical) analgesia was similar between the groups. Non-medical analgesia methods (including bath, TENS, etc.) were more frequently used in the non-GDM group. Both groups had low rates of women who delivered completely without analgesia (0.4% vs 0.6%).

**Table 1:** Use of labor analgesia in parturients with gestational diabetes mellitus (GDM) and without GDM in Finland from 2004 to 2018

	GDM		non-GDM	
	*n*	%	*n*	%
Epidural	44,450	47.8	135,904	49.9
Spinal	15,618	16.8	43,163	15.9
Combined spinal–epidural	2,387	2.6	5,797	2.1
Paracervical block	15,839	17.0	45,947	16.9
Pudendal block	9,067	9.8	25,873	9.5
Nitrous oxide	53,761	57.8	158,815	58.3
Other medical analgesia	11,947	12.9	36,317	13.3
Other non-medical analgesia	29,245	31.5	93,999	34.5
No analgesia	551	0.6	1,152	0.4

## Discussion

Based on the results of this nationwide register-based study, parturients with GDM seem to have similar analgesia usage as parturients without GDM. Minor difference was observed in the use of epidural analgesia, but this finding was not clinically relevant as the overall use of neuraxial analgesia was similar between the two groups. Furthermore, we report that the GDM testing rate has been increasing continuously as well as the number of GDM patients, even though overall delivery rate has been decreasing in recent years in Finland.

Compared to a previous report which described increased opioid consumption after cesarean section, our report focused on intrapartum analgesia, and we did not find any meaningful differences between women with and without GDM [3]. We excluded elective cesarean sections as the mode of the anesthesia is not reported to the register. Furthermore, we had no information on postpartum/postoperative analgesia, so therefore our results are not comparable to Yang et al. study [3]. A previous Finnish study compared labor analgesia between obese and non-obese parturients and had similar results [4]. Thus, it seems that parturients with either maternal obesity or GDM have similar labor analgesia usage as other parturients.

The main strength of the present study is the nationwide register coverage including practically all deliveries in Finland and the high validity and precision of the register. Furthermore, the diagnostic and the testing criteria for GDM remained unchanged during our follow-up period. The main limitation is the lack of data on attempted analgesia methods. For example, some part of the slightly lower epidural rate in the GDM group could be due to unsuccessful attempts, as only successful analgesia methods are reported to the register. Another limitation is that the register does not have information on analgesic doses and therefore possible differences between the two groups remain unknown. Furthermore, the register only gathers information on intrapartum analgesia; hence, we have not analyzed postpartum analgesia. Another clear limitation is the lack of glycemic control information and therapies used to treat GDM as these were not reported to the register during the study period.

In conclusion, we found that parturients with GDM have similar labor analgesia than parturients without GDM. This finding is important for clinicians and guideline makers as the number of parturients with GDM has been increasing.
